# Comparison of characteristics between Chinese diabetes mellitus-induced erectile dysfunction populations and non-diabetes mellitus-induced erectile dysfunction populations: A cross-sectional study

**DOI:** 10.3389/fendo.2022.1096045

**Published:** 2022-12-21

**Authors:** Jingxuan Peng, Dongjie Li, Longyun Liu, Yali Xiang, Yuxin Tang

**Affiliations:** ^1^ Department of Urology, Xiangya Hospital, Central South University, Changsha, Hunan, China; ^2^ National Clinical Research Center for Geriatric Disorders, Xiangya Hospital, Central South University, Changsha, China; ^3^ Department of Urology, First Affiliated Hospital of Jishou University, Jishou, Hunan, China; ^4^ Department of Urology, The Fifth Affiliated Hospital, Sun Yat-sen University, Zhuhai, China; ^5^ Guangdong Provincial Key Laboratory of Biomedical Imaging, The Fifth Affiliated Hospital, Sun Yat-sen University, Zhuhai, China; ^6^ Health Management Center, The Fifth Affiliated Hospital of Sun Yat-sen University, Zhuhai, China

**Keywords:** erectile dysfunction, diabetes mellitus, influence factors, cross-sectional study, clinical study

## Abstract

**Background:**

Erectile dysfunction (ED) is a common disease in adult men, and diabetes is an independent risk factor for ED. However, there are few reports on the distinction between diabetes mellitus-induced erectile dysfunction (DMED) and non-DMED features, as well as ED features of varying severity in the two groups.

**Methods:**

A total of 365 ED patients treated at two clinics in China from 2019 to 2022 were included. Questionnaires of the International Index of Erectile Function (IIEF-5), Erectile Hardness Score (EHS), Premature Ejaculation Diagnostic Tool (PEDT), Patient Health Questionnaire-9 (PHQ-9), and Generalized Anxiety Disorder-7 (GAD-7) were administered to the patients. They were divided into three groups according to the IIEF-5 score: 5-7 for severe ED, 8-11 for moderate ED, and 12-21 for mild ED. In addition, the patient’s age, weight, height, fasting blood glucose (FBG), total cholesterol (TC), triglycerides (TG), follicle-stimulating hormone (FSH), luteinizing hormone (LH), prolactin (PRL), total testosterone (TT) and other indicators were also collected. Statistical analysis was performed using SPSS 26, comparing all parameters between groups.

**Results:**

Age (P<0.001), height (P=0.009), body mass index (BMI) (P=0.002), PEDT (P<0.001), FBG (P<0.001), FSH (P<0.001), TG (P<0.001), TT (P<0.001) and triglyceride-glucose index (TyG) (P<0.001) were significantly different between diabetic ED and nondiabetic ED subjects. The trend test in the nondiabetic ED population found a negative correlation between the IIEF-5 score and PHQ-9 (P for trend=0.15). Multivariate ordinal logistic regression in the diabetic ED population showed that elevated LH OR=11.37 (95% CI: 0.966, 3.897) and elevated PRL OR=4.10 (95% CI: 0.410, 2.411) were associated with an increased risk of more severe ED.

**Conclusions:**

The aetiology, demographic parameters, degree of premature ejaculation, and related biochemical tests were significantly different between the DMED and non-DMED populations.

## Introduction

Erectile dysfunction (ED) is a common disease in adult men, and diabetes is one of the clear independent risk factors for ED (1). Diabetes mellitus erectile dysfunction (DMED) patients tend to have more severe symptoms, a worse curative effect, a greater impact on their quality of life, and a serious impact on their marital relationship and even their social harmony (2, 3). The pathogenesis of DMED is extremely complex, and studies have shown that damage to smooth muscle cells in the cavernous sinus and endothelial cell dysfunction caused by hyperglycaemia are its core mechanisms (4, 5). Hyperglycaemia can affect the normal function of mitochondria in diabetic patients and stimulate oxidative stress. Excessive oxidative stress can lead to damage and dysfunction of the smooth muscle cells and vascular endothelial cells in the corpus cavernosum, which in turn affects erectile function (5–7). Phosphodiesterase type 5 inhibitors (PDE-5Is) are currently the first-line regimen for ED treatment (6), but clinically, it has been found that they are less effective in DMED patients (7, 8).

In fact, compared with non-DMED patients, DMED patients not only have a higher incidence of ED (twice the rate of non-DMED) but are also characterized by worse treatment effects and more severe erectile dysfunction (8–11). Therefore, clarifying the difference between the two is crucial for improving ED diagnosis and treatment. However, differences in the characteristics of DMED and non-DMED, as well as differences in ED-related characteristics of different severities in diabetic patients, have rarely been reported (12).

Therefore, this study intended to explore the characteristics and differences between the DMED and the non-DMED populations among Chinese male patients attending an andrology outpatient clinic, which is of great significance for improving ED diagnosis and treatment (13–15).

## Materials and methods

### Participants

This study was designed as a cross-sectional study and was approved by the local ethics committees. All patients gave informed consent according to the Institutional Review Board guidelines and the Declaration of Helsinki. All protocols were approved by the institutional review boards of Xiangya Hospital of Central South University (No. 201904092) and the Fifth Affiliated Hospital of Sun Yat-Sen University (No. 2019-K231).

The consecutive enrolment method was used to recruit patients who visited the andrology outpatient clinics of Xiangya Hospital of Central South University and the Fifth Affiliated Hospital of Sun Yat-sen University from 2019 to 2022. Informed consent was obtained, and interviews could be interrupted or withdrawn from at any time. Inclusion criteria for the diabetic ED group: 1. History of diabetes; 2. The IIEF-5 score suggests ED; 3. No other ED-related diseases; 4. Willingness to participate in this study. Inclusion criteria for the nondiabetic ED group: 1. No history of diabetes; 2. The IIEF-5 score suggests ED; 3. No other diseases; 4. Willingness to participate in this study. According to the above criteria, a total of 365 participants were included in this study, including 135 patients with diabetic ED (91 mild, 26 moderate and 18 severe) and 230 nondiabetic ED patients (146 mild, 60 moderate and 24 severe).

### Measures

#### Classification of ED according to IIEF-5

All participants were administered the IIEF-5 questionnaire, underwent a physical examination and provided a medical history to diagnose ED. The patients were divided into three groups according to symptom severity. Patients with an IIEF-5 score of 21–25 points were included in the mild ED group, patients with an IIEF-5 score of 11–20 points were included in the moderate ED group, and patients with an IIEF-5 score of 5–10 points were included in the severe ED group.

### Laboratory analysis

Fasting blood glucose (FBG), total cholesterol (TC), triglycerides (TG), total testosterone (TT), follicle-stimulating hormone (FSH), prolactin (PRL) and luteinizing hormone (LH) of all subjects were obtained from the hospital laboratory. Normal range: FBG: 3.9-6.1 mmol/L, TC: <5.18 mmol/L, TG: <1.7 mmol/L, TT: 8.64-29.0 mmol/L, FSH: 1.5-12.4 mmol/L, PRL: 4.04-15.02 mmol/L and LH: 1.7-8.6 mmol/L. The triglyceride-glucose index (TyG), developed by Simental-Mendía et al. (16), was calculated from the fasting serum glucose and triglyceride values.

### Related scales

Sexual function was assessed by andrology-related scales, including the IIEF-5, EHS, and PEDT. The erection hardness was evaluated using the EHS: penis is larger but not hard (I), penis is hard but not hard enough for penetration (II), penis is hard enough for penetration but not completely hard (III), and penis is completely hard and fully rigid (IV). Premature ejaculation (PE) was diagnosed by the PEDT: PE (≥11), suspected PE (9−10), and non-PE (≤8).

The patients were also asked to complete the GAD-7, which was used to assess anxiety symptoms, as well as the PHQ-9, which was used to evaluate depressive symptoms.

### Questionnaire validity

The Cronbach’s alpha score was calculated as 0.78, showing adequate internal consistency. The test-retest correlation coefficients of each item were ≥0.77, indicating excellent stability over time (P < 0.001) (**Table 1**).

**Table 1:** Test−retest correlation coefficients (R) and P values of IIEF-5, PEDT, PHQ-9, EHS and GAD-7.

	IIEF-5					PEDT				
Question	1	2	3	4	5	1	2	3	4	5
R	0.785	0.773	0.777	0.779	0.791	0.773	0.770	0.771	0.766	0.770
P	<0.001	<0.001	<0.001	<0.001	<0.001	<0.001	<0.001	<0.001	<0.001	<0.001
	**PHQ-9**									**EHS**
Question	1	2	3	4	5	6	7	8	9	
R	0.771	0.768	0.772	0.767	0.775	0.766	0.773	0.771	0.776	0.781
P	<0.001	<0.001	<0.001	<0.001	<0.001	<0.001	<0.001	<0.001	<0.001	<0.001
	**GAD-7**									
Question	1		2	3	4		5	6		7
R	0.767		0.769	0.771	0.771		0.769	0.766		0.771
P	<0.001		<0.001	<0.001	<0.001		<0.001	<0.001		<0.001

### Statistical analysis

Data are expressed as the mean ± SD for normally distributed parameters and as the median (quartile) for nonnormally distributed parameters. When normal and nonnormal distributions were used, correlations were performed using Student’s t test and the Wilcoxon rank-sum (Mann−Whitney U) test, respectively. Univariable and multivariable ordinal logistic regression analyses were used for multivariate analysis and continuous or categorical dependent variables, respectively. ANOVA was used to assess the differences in the clinical variables between the two populations. We used G-Power software to verify the sample size of the study. Under the assumption of an effect size of 0.5, α=0.5, and a test power of 0.95, the sample size needed to be greater than 42 to effectively verify the conclusion. All statistical analyses were performed using SPSS Statistics (IBM, version 26.0; SPSS, Armonk, NY, USA) for Windows. P < 0.05 was considered to indicate statistical significance.

## Results

### Comparison of the characteristics of the two populations

**Table 2** shows the results of the main sociodemographic and biochemical characteristics and related scales of the participants with DMED and non-DMED. Among them, 135 patients with DMED and 230 patients with non-DMED were included in this study. In our study, age (P<0.001), height (P=0.009), BMI (P=0.002), PEDT (P<0.001), FBG (P<0.001), FSH (P<0.001), TG (P < 0.001), TT (P < 0.001) and TyG (P < 0.001) were significantly different between the two groups.

**Table 2:** Comparison of the Characteristics between the Two Groups (DMED vs Non-DMED).

	DMED	Non-DMED	t	P
**Age**	**43.79 ± 8.91**	**33.48 ± 7.95**	**11.09**	**<0.001**
**Height**	**168.62 ± 5.79**	**170.30 ± 5.41**	**-2.76**	**0.006**
Weight	69.16 ± 8.41	67.78 ± 9.10	1.43	0.153
**BMI**	**24.32 ± 2.62**	**23.36 ± 2.89**	**3.20**	**0.002**
IIEF-5	13.56 ± 4.76	14.05 ± 4.71	-0.95	0.341
EHS	2.59 ± 0.77	2.56 ± 0.80	0.36	0.717
**PEDT**	**13.37 ± 4.92**	**17.76 ± 5.08**	**-7.98**	**<0.001**
PHQ9	6.46 ± 4.18	6.90 ± 4.98	-0.87	0.387
GAD7	6.32 ± 4.15	5.52 ± 4.55	1.61	0.098
**FBG**	**7.70 ± 2.10**	**5.17 ± 0.39**	**13.56**	**<0.001**
TC	4.78 ± 1.96	4.84 ± 1.06	-0.31	0.760
**FSH**	**5.29 ± 2.54**	**4.36 ± 2.10**	**3.63**	**<0.001**
**TG**	**2.31 ± 1.43**	**1.66 ± 0.93**	**4.60**	**<0.001**
PRL	13.32 ± 5.12	13.45 ± 8.75	-0.17	0.869
LH	5.28 ± 2.34	5.06 ± 2.01	0.90	0.368
**TT**	**14.38 ± 5.12**	**18.77 ± 6.65**	**-6.76**	**<0.001**
**TyG**	**7.76 ± 0.59**	**7.08 ± 0.52**	**10.63**	**<0.001**

### Internal feature comparisons

EHS (P<0.001) and TC (P=0.022) were significantly different among the three groups with different degrees of DMED (**Table 3.1**). GAD7 (P=0.048) and FBG (P=0.032) were significantly different in mild and moderate cases. TT (P=0.031) was only significantly different between the moderate and severe groups.

**Table 3.1:** The Results of LSD Among Mild ED, Moderate ED and Severe ED in DMED Populations.

	Mild ED	Moderate ED	Severe ED	F	P
Age	43.55 ± 8.62	45.89 ± 10.69	41.94 ± 7.36	1.139	0.323
Height	168.57 ± 6.09	167.54 ± 4.17	170.44 ± 6.06	1.360	0.260
Weight	70.50 ± 9.03	69.37 ± 14.31	71.61 ± 10.91	0.251	0.779
BMI	24.79 ± 2.71	24.68 ± 4.58	24.59 ± 3.09	0.035	0.966
EHS	2.71 ± 0.62*	2.50 ± 0.95#	1.83 ± 0.99*#	10.55	**<0.001**
PEDT	13.40 ± 4.68	13.72 ± 5.14	12.78 ± 5.91	0.193	0.825
PHQ9	6.29 ± 3.67	6.08 ± 3.77	7.76 ± 6.42	0.993	0.374
GAD7	5.71 ± 3.49 #	7.60 ± 5.01#	7.18 ± 5.09	2.426	0.093
FBG	7.49 ± 1.65#	8.52 ± 3.31#	7.55 ± 2.10	2.402	0.095
TC	4.51 ± 1.22#	5.68 ± 3.31#	4.68 ± 1.91	3.912	**0.022**
FSH	5.40 ± 2.63	5.29 ± 3.01	4.95 ± 2.04	0.219	0.803
TG	2.36 ± 1.54	1.96 ± 1.15	2.43 ± 1.14	0.892	0.412
PRL	13.22 ± 4.96	12.63 ± 4.76	14.56 ± 5.20	0.836	0.436
LH	5.21 ± 2.27	5.45 ± 2.47	5.41 ± 2.62	0.137	0.872
TT	14.53 ± 5.27	15.46 ± 4.36 #	12.08 ± 4.95#	2.497	0.086
TyG	7.77 ± 0.57	7.66 ± 0.76	7.76 ± 0.59	0.688	0.500

#P<0.05, *P<0.001.

EHS (P < 0.001) and PHQ9 (P = 0.011) were significantly different among the three groups with different degrees of non-DMED (**Table 3.2**). Subsequently, the correlation analysis of IIEF-5 and PHQ9 found a negative correlation between the two (P *_for trend_* =0.15). PRL (P=0.016) was significantly different between patients with moderate and severe disease.

**Table 3.2:** The Results of LSD Among Mild ED, Moderate ED and Severe ED in Non-DMED Populations.

	Mild ED	Moderate ED	Severe ED	F	P
Age	33.32 ± 7.58	33.27 ± 7.94	35.00 ± 10.14	0.487	0.615
Height	170.27 ± 5.47	170.40 ± 4.80	170.22 ± 6.65	0.016	0.984
Weight	67.23 ± 9.05	69.95 ± 8.80	65.78 ± 9.53	2.464	0.087
BMI	23.18 ± 2.88	24.09 ± 2.85	22.69 ± 2.85	2.711	0.069
EHS	2.83 ± 0.66^*1*2^	2.22 ± 0.83^*1#^	1.78 ± 0.74^*2#^	**30.442**	**<0.001**
PEDT	17.85 ± 4.89	17.36 ± 5.39	18.26 ± 5.65	0.320	0.727
PHQ9	**6.16 ± 4.37^#1^**	**8.05 ± 5.35 ^#2^**	**8.50 ± 6.60 ^#1#2^**	**4.606**	**0.011**
GAD7	5.05 ± 4.14	6.32 ± 4.88	6.33 ± 5.74	2.104	0.124
FBG	5.18 ± 0.39	5.19 ± 0.34	5.09 ± 0.57	0.345	0.709
TC	4.82 ± 1.08	4.84 ± 0.85	4.94 ± 1.46	0.082	0.921
FSH	4.34 ± 2.23	4.38 ± 1.76	4.50 ± 2.10	0.046	0.955
TG	1.66 ± 0.92	1.69 ± 0.97	1.62 ± 0.91	0.032	0.968
PRL	13.47 ± 8.59	11.95 ± 6.18 **^#^**	17.82 ± 14.19 **^#^**	2.935	0.055
LH	5.11 ± 2.20	4.91 ± 1.47	5.15 ± 1.94	0.202	0.818
TT	19.19 ± 6.42	18.33 ± 7.59	16.86 ± 5.12	1.074	0.344
TyG	7.07 ± 0.52	7.09 ± 0.51	7.07 ± 0.61	0.019	0.981

#P<0.05, *P<0.001.

### Logistic regression results for the two populations

Univariate and multivariate ordinal logistic regression analyses were performed on various factors that may affect the degree of ED in the DMED populations, and EHS (P<0.001) and total cholesterol were included as covariates (TT was normal in the DMED population and thus not included in the comparison).

Univariate ordinal logistic regression (**Table 4.1**) showed that major depression OR=7.32 (95% CI: 0.271, 3.710) was associated with an increased risk of more severe ED. Multiple ordinal logistic regression showed that increased LH OR=11.37 (95% CI: 0.966, 3.897) and PRL OR=4.10 (95% CI: 0.410, 2.411) were associated with an increased risk of more severe ED.

**Table 4.1:** Univariable and multivariable ordinal logistic regression analysis among DMED Populations.

	Univariable OR (95% CI)	P	Multivariable OR (95% CI)	P
Age				
20-29	Ref		Ref	
30-39	2.83 (-1.268,3.350)	0.377	1.61 (-2.181,3.133)	0.726
40-49	1.29 (-2.095,2.609)	0.830	3.26 (-1.490,3.852)	0.386
50-59	2.33 (-1.495,3.185)	0.479	3.00 (-1.601,3.802)	0.425
>60	3.04 (-1.625,3.847)	0.426	1.86 (-2.759,3.994)	0.720
	*P for trend*	0.520	*P for trend*	0.899
PEDT				
Non-PE	Ref		Ref	
Suspected PE	0.659 (-1.589,0.725)	0.464	1.09 (-1.791,1.958)	0.930
PE	0.46 (-1.678,0.141)	0.098	0.35 (-2.464,0.384)	0.152
	*P for trend*	0.363	*P for trend*	0.499
PHQ-9				
No depression	Ref		Ref	
Mild depression	0.62 (-1.310,0.362)	0.267	0.56(-2.081,0.917)	0.446
Moderate depression	0.78 (-1.624,1.135)	0.728	0.15 (-4.014,0.200)	0.076
Severe depression	**7.32 (0.271,3.710)**	**0.023**	2.25 (-1.726,3.352)	0.530
	*P for trend*	0.072	*P for trend*	0.156
GAD-7				
No anxiety	Ref		Ref	
Mild anxiety	0.67 (-1.276,0.472)	0.367	0.80 (-1.723,1.281)	0.773
Moderate anxiety	2.45 (-0.145,1.936)	0.092	7.54 (-0.059,4.099)	0.057
Severe anxiety	3.46 (-0.941,3.424	0.265	10.36(-0.340,5.061)	0.087
	*P for trend*	0.250	*P for trend*	0.419
FBG				
Normal	Ref		Ref	
Elevated	0.41 (-2.174,0.389)	0.172	0.23 (-3.012,0.068)	0.061
	*P for trend*	0.187	*P for trend*	0.077
TG				
Normal	Ref		Ref	
Elevated	0.95 (-0.763,0.662)	0.889	0.64 (-1.440,0.563)	0.391
	*P for trend*	0.050	*P for trend*	0.050
FSH				
Normal	Ref		Ref	
Elevated	0.60 (-2.848,1.838)	0.673	1.53 (-3.811,4.664)	0.884
	*P for trend*	0.598	*P for trend*	0.883
LH				
Normal	Ref		Ref	
Elevated	1.69 (-0.498,1,552)	0.314	**11.37 (0.966,3.897)**	**0.001**
	*P for trend*	0.556	*P for trend*	0.816
PRL				
Normal	Ref		Ref	
Elevated	1.36 (-0.412,1.033)	0.399	**4.10 (0.410,2.411)**	**0.006**
	*P for trend*	0.867	*P for trend*	0.495

Univariate and multivariate ordinal logistic regression analyses were performed on various factors that may affect the degree of non-DMED, and EHS (P<0.001) and PHQ9 (P=0.011) were included as covariates (FBG, FSH and TT were normal in the non-DMED populations and thus were not included in the comparison).

Univariate logistic regression (**Table 4.2**) showed that severe anxiety OR=0.26 (95% CI: -2.473, -0.227) was associated with an increased risk of more severe ED. However, this result was not observed in the multivariate logistic regression results.

**Table 4.2:** Univariable and multivariable ordinal logistic regression analysis among Non-DMED Populations.

	Univariable OR (95% CI)	P	Multivariable OR (95% CI)	P
Age				
20-29	Ref		Ref	
30-39	0.81 (-0.821,0.395)	0.492	1.19 (-0.642,0.981)	0.682
>40	1.02 (-0.737,0.784)	0.951	2.01 (-0.364,1.758)	0.198
	P for trend	0.156	P for trend	0.948
PEDT				
Non-PE	Ref		Ref	
Suspected PE	0.61 (-1.676,1.539)	0.993	2.81 (-0.913,2.977)	0.298
PE	0.57 (-0.636,1.616)	0.395	0.84 (-1.688,1.333)	0.818
	P for trend	0.304	P for trend	0.599
GAD-7				
No anxiety	Ref		Ref	
Mild anxiety	0.81 (-0.802,0.374)	0.475	0.82 (-1.056,0.650)	0.641
Moderate anxiety	0.81 (-1. 080,0.666)	0.642	2.20 (-0.572,2.146)	0.257
Severe anxiety	0.26 (-2.473, -0.227)	**0.019**	0.28(-2.974,0.433)	0.144
	P for trend	0.266	P for trend	0.145
TG				
Normal	Ref		Ref	
Elevated	0.91 (-0.699,0.518)	0.771	0.51 (-1.445,0.089)	0.083
	P for trend	0.979	P for trend	0.864
TC				
Normal	Ref		Ref	
Elevated	0.87 (-0.773,0.486)	0.656	1.88 (-0.161,1.422)	0.118
	P for trend	0.438	P for trend	0.402
LH				
Normal	Ref		Ref	
Elevated	0.99 (-1.391,1.380)	0.995	2.42 (-0.846,2.615)	0.317
	P for trend	0.679	P for trend	0.971
PRL				
Normal	Ref		Ref	
Elevated	1.18 (-0.502,0.832)	0.628	1.15 (-0.664,0.953)	0.727
	P for trend	0.747	P for trend	0.792

## Discussion

ED is one of the most common disorders in andrology clinics, with an estimated prevalence between 30% and 50% in the general population (17–20). As the number of people with diabetes worldwide is increasing, there is growing concern about erectile dysfunction in men with diabetes (19–21). At present, although there are many studies on DMED, comparisons of the characteristics of DMED and non-DMED populations, especially the differences in ED-related characteristics among diabetic patients with varying severity, are rarely reported. Therefore, this study fills this gap, which is of great significance for improving ED diagnosis and treatment.

This study compared the sociodemographic and biochemical characteristics and related scales of the DMED and non-DMED populations after assessing the validity of the questionnaire. The results showed that age (43.79 ± 8.91 vs. 33.48 ± 7.95, P<0.001), height (168.62 ± 5.79 vs. 170.30 ± 5.41, P=0.006), BMI (24.32 ± 2.62 vs. 23.36 ± 2.89, P=0.002), PEDT (13.374 ± 4.92 vs. 17.76 ± 5.08, P<0.001), FBG (7.70 ± 2.10 vs. 5.17 ± 0.39, P<0.001), FSH (5.29 ± 2.54 vs. 4.36 ± 2.10, P<0.001), TG (2.31 ± 1.43 vs. 1.66 ± 0.93, P < 0.001), TT (14.38 ± 5.12 vs. 18.77 ± 6.65, P < 0.001) and TyG (7.76 ± 0.59 vs. 7.08 ± 0.52, P < 0.001) were significantly different between the two groups. **Table 3.2** indicates that the non-DMED population in this study mainly has psychogenic ED, and the regression analysis of the two populations suggests that the DMED populations mainly have organic ED. Thus, non-DMED patients were younger and had higher PEDT scores. In addition, studies have shown that diabetes not only causes increased FSH and atherosclerosis leading to increased TG but also leads to decreased serum TT levels and insulin resistance (higher TyG) (22, 23), which was also verified in this study. However, research on the underlying mechanism is rarely reported, which may be the direction of our future research (24).

In the internal comparison within the groups by symptom severity, EHS (P<0.001) and total cholesterol (P=0.022) were found to be significantly different in the three groups with different degrees of DMED. EHS (P<0.001) and PHQ9 (P=0.011) were significantly different in the three groups with different degrees of non-DMED, and the trend test found that the IIEF-5 score and PHQ9 were negatively correlated (P for trend=0.15), which suggests that psychogenic ED is predominant in non-DMED patients.

Finally, univariate and multivariate ordinal logistic regression analyses were performed on various factors that may affect the degree of ED among DMED and non-DMED populations, respectively. In the DMED population, univariate ordinal logistic regression showed that severe depression OR=7.32 (95% CI: 0.271, 3.710) was associated with an increased risk of severe ED. Multiple ordinal logistic regression showed that increased LH OR=11.37 (95% CI: 0.966, 3.897) and PRL OR=4.10 (95% CI: 0.410, 2.411) were associated with an increased risk of severe ED. In the non-DMED population, univariate logistic regression showed that severe anxiety OR=0.26 (95% CI: -2.473, -0.227) was associated with an increased risk of more severe ED, but no correlations were found in the multivariate logistic regression. A comparison of the two groups showed that increased LH and PRL were associated with an increased risk of more severe ED in the DMED population. This can be attributed to diabetes-induced metabolic syndrome leading to elevated LH and hyperprolactinemia (HyperPRL) causing ED, and the related mechanisms are currently being studied (25–31). Therefore, screening and appropriate interventions for men with erectile dysfunction is warranted (32). Our findings suggest that early detection, early diagnosis and early treatment of diabetes are essential to prevent the occurrence of ED, and standardized treatment of diabetes is of great significance for improving erectile function.

This study has some merits and shortcomings. The main merit is this study was carried out in two top clinics in China, with highly professional staff, a sufficient number of patients and advanced equipment. Meanwhile, this study has the following limitations, which need to be resolved in future studies. First, objective measurements (night time penile erection monitoring equipment and Doppler ultrasound equipment) can evaluate ED more reliably than questionnaire surveys, but related studies have also confirmed the effectiveness of IIEF questionnaires for ED evaluation (33). The main basis of this study was using the IIEF-5 questionnaire, a physical examination and the medical history to diagnose ED cases and stratify them by severity. Second, this investigation was an observational cross-sectional study, and no longitudinal data were available for the interaction of diabetes and erectile function. Therefore, relevant studies, including longitudinal studies and treatments, are needed in the future to assess changes in erectile function during diabetes treatment and this may provide new insights into treatment strategies for ED.

In conclusion, there are significant differences in aetiology between diabetic ED and nondiabetic ED patients. Nondiabetic ED patients mainly have psychogenic ED, while diabetic ED patients mainly have organic ED. In addition, there are differences in demographic parameters such as age, height, weight, BMI, biochemical tests such as blood glucose, triglycerides, total testosterone, and PEDT. Therefore, individualized treatment according to the characteristics of the different populations is of great significance for improving erectile function.

## Data availability statement

The raw data supporting the conclusions of this article will be made available by the authors, without undue reservation.

## Ethics statement

The studies involving human participants were reviewed and approved by Xiangya Hospital of Central South University (No. 201904092) and the Fifth Affiliated Hospital of Sun Yat-Sen University (No. 2019-K231). The patients/participants provided their written informed consent to participate in this study.

## Author contributions

DL and YT designed the experiments. JP and LL contributed to the clinical data collection and assessment. JP, DL and YX analysed the results. JP, DL and YT wrote the manuscript. All authors contributed to the article and approved the submitted version.
